# Occipital lymph node metastasis from nasopharyngeal carcinoma: a special case report and literature review

**DOI:** 10.1186/s40880-015-0074-y

**Published:** 2016-01-04

**Authors:** Jing Yang, Wei-Xiong Xia, Yan-Qun Xiang, Xing Lv, Liang-Ru Ke, Ya-Hui Yu, Xiang Guo

**Affiliations:** Department of Nasopharyngeal Carcinoma, State Key Laboratory of Oncology in South China, Collaborative Innovation Center for Cancer Medicine, Sun Yat-sen University Cancer Center, 651 Dongfeng Road East, Guangzhou, 510060 Guangdong P.R. China

**Keywords:** Nasopharyngeal carcinoma, Occipital lymph node, Lymphatic metastasis, Chemoradiotherapy, Intensity-modulated radiation therapy

## Abstract

Cervical lymph node metastasis is common in patients with nasopharyngeal carcinoma (NPC), but occipital lymph node metastasis in NPC patients has not yet been reported. In this case report, we describe an NPC patient with occipital lymph node metastasis. The clinical presentation, diagnostic procedure, treatment, and outcome of this case were presented, with a review of the related literature.

## Background

In South China, nasopharyngeal carcinoma (NPC) is a common head and neck cancer, with an incidence of 15–50 per 100,000 people [1, 2]. It is often called “Canton Tumor” because of the highest morbidity of NPC in Guangdong Province, China. More than 70% of NPC patients have already developed cervical lymph node metastasis at initial diagnosis [3, 4]. Based on the American Joint Committee on Cancer classification [5], the definition of the cervical levels is shown in Table 1. Cervical levels of NPC, from high to low incidence, are level II, level III, level V, level IV, supraclavicular region, level I, and level VI [3]. However, to our knowledge, metastasis to the occipital lymph node in NPC has not been reported. In this case report, we describe one NPC patient with occipital lymph node metastasis and discuss the treatment regimen. We also review related literature.

**Table 1:** Anatomical structures defining the boundaries of the cervical levels and sublevels

Boundary level	Superior	Inferior	Anterior (medial)	Posterior (lateral)
IA	Symphysis of mandible	Body of hyoid	Anterior belly of contralateral digastric muscle	Anterior belly of ipsilateral digastric muscle
IB	Body of mandible	Posterior belly of diagastric muscle	Anterior belly of digastric muscle	Stylohyoid muscle
IIA	Skull base	Horizontal plane defined by the inferior border of the hyoid bone	The stylohyoid muscle	Vertical plane defined by the spinal accessory nerve
IIB	Skull base	Horizontal plane defined by the inferior body of the hyoid bone	Vertical plane defined by the spinal accessory nerve	Lateral border of the sternocleidomastoid muscle
III	Horizontal plane defined by the inferior body of hyoid	Horizontal plane defined by the inferior border of the cricoid cartilage	Lateral border of the sternohyoid muscle	Lateral border of the sternocleidomastoid or sensory branches of cervical plexus
IV	Horizontal plane defined by the inferior border of the cricoid cartilage	Clavicle	Lateral border of the sternohyoid muscle	Lateral border of the sternocleidomastoid or sensory branches of cervical plexus
VA	Apex of the convergence of the sternocleidomastoid and trapezius muscles	Horizontal plane defined by the lower border of the cricoid cartilage	Posterior border of the sternocleidomastoid muscle or sensory branches of cervical plexus	Anterior border of the trapezius muscle
VB	Horizontal plane defined by the lower border of the cricoids cartilage	Clavicle	Posterior border of the sternocleidomastoid muscle	Anterior border of the trapezius muscle
VI	Hyoid bone	Suprasternal notch	Common carotid artery	Common carotid artery
VII	Suprasternal notch	Innominate artery	Sternum	Trachea, esophagus, and prevertebral fascia

## Case report

A 19-year-old man from Jiangxi Province, China, was admitted with the chief complaint of bilateral cervical masses for 3 months. The patient did not complain any of the following symptoms: fever, nose bleeding, obstruction, tinnitus, diplopia, or headache. He had no history of trauma, surgery, smoking, or drinking. Physical examination showed a neoplasm in the nasopharynx and several enlarged cervical lymph nodes of bilateral levels II–V; the largest one was 10 cm × 8 cm. In addition, an occipital lymph node of 2 cm × 2 cm was palpable, with medium firmness and clear edge. The laboratory results were normal except for the results of Epstein-Barr virus (EBV). EBV levels were abnormally elevated: EBV viral capsid antigen (VCA)-IgA, 1:640; EBV early antigen (EA)-IgA, 1:40; and EBV-DNA, 8.82 × 10^5^ copies/mL.

**Fig. 1:**
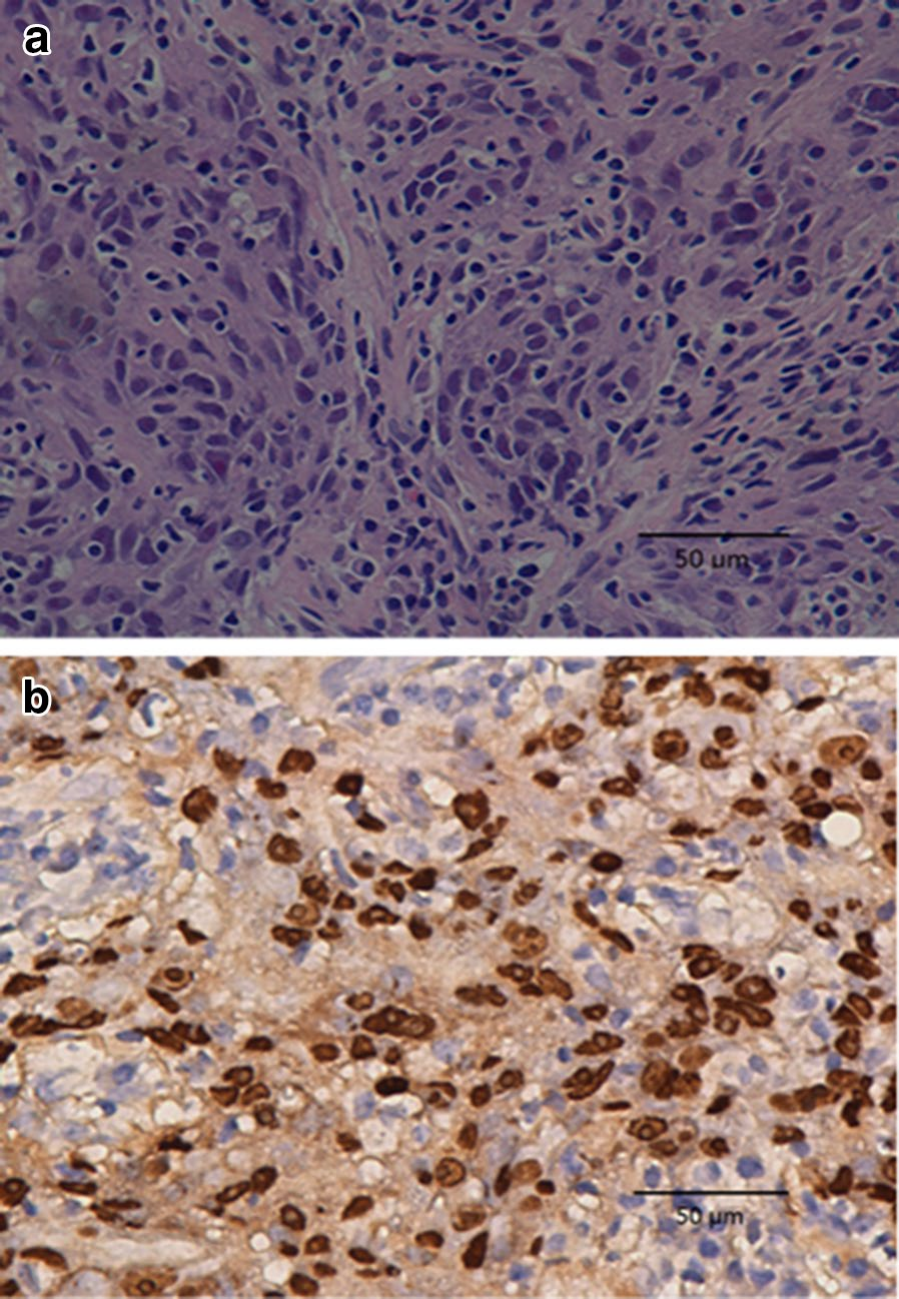
Representative images of pathologic slices from fine-needle aspiration of the occipital lymph node (original magnification, ×40). **a** Hematoxylin and eosin (H and E) stained section shows diffused tumor cells displaying characteristics of nasopharyngeal carcinoma (NPC) cells. **b** Immunohistochemical analysis and in situ hybridization of the occipital lymph node shows the expression of Epstein-Barr virus-encoded RNAs (EBERs) in tumor cells

Biopsy of the nasopharyngeal neoplasm confirmed undifferentiated non-keratinizing carcinoma. Because of the possibility, though rare, of NPC-caused metastasis in the occipital region, a fine-needle aspiration of the occipital lymph node was also performed. The pathologic report after hematoxylin and eosin (H and E) staining identified poorly differentiated carcinoma, which suggested metastasis from NPC (Fig. 1a). Immunohistochemical analysis and in situ hybridization of the occipital lymph node further confirmed the presence of EBV-encoded RNAs (EBERs), indicating EBERs expression in tumor cells (Fig. 1b).

**Fig. 2:**
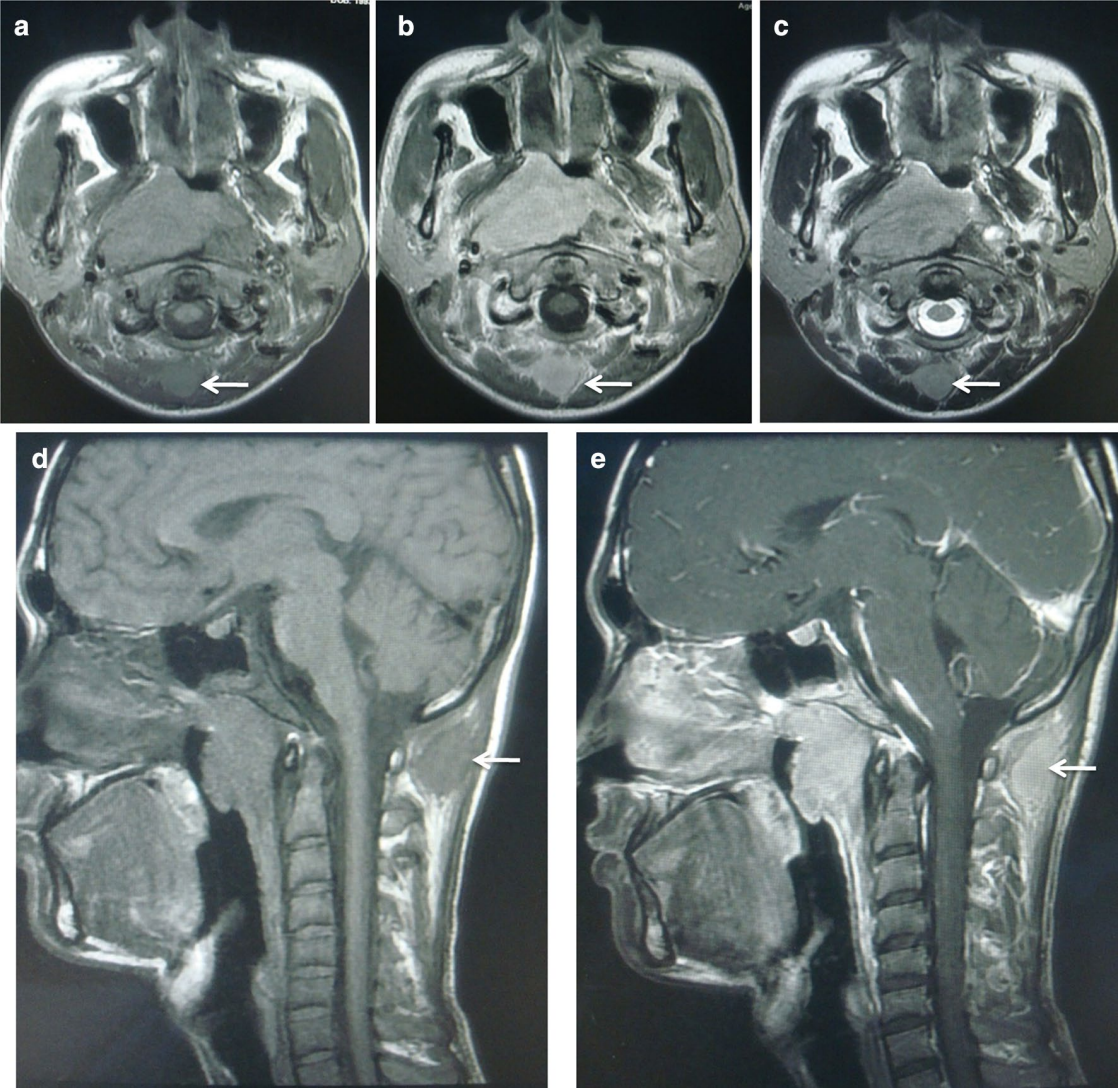
Magnetic resonance (MR) imaging of the NPC patient before treatment. T1-weighted axial MR images **a** without contrast, **b** with contrast, and **c** T2-weighted axial MR image show an occipital lymph node (18 mm × 19 mm) with equal T1 signal, long or equal T2 signal, and obvious enhancement (*arrows*). T1-weighted sagittal MR image **d** without contrast and **e** with contrast also show an enlarged lymph node with enhancement in subcutaneous tissue of the occiput (*arrows*)

Magnetic resonance imaging (MRI) of the nasopharynx and neck revealed that the tumor extended into the right parapharyngeal space, right carotid sheath, right medical pterygoid muscle, right pterygopalatine fossa, right cavernous sinus, and vast area of the skull base. Bilaterally enlarged retropharyngeal lymph nodes and cervical lymph nodes at level IIa, IIb, III, IV, Va, and Vb were also detected. An occipital lymph node of 18 mm × 19 mm was detected by MRI (Fig. 2). Other radiographic studies, including chest radiography, abdominal sonography, and a bone scan, showed no distant metastasis. Accordingly, the patient was diagnosed comprehensively as having T4N3M0 stage IVb undifferentiated non-keratinizing NPC.

The patient received two cycles of neoadjuvant chemotherapy with cisplatin (80 mg/m^2^, day 1) and 5-fluorouracil (4000 mg/m^2^, days 1–5), repeated every 21 days. The patient also received two cycles of concurrent cisplatin (80 mg/m^2^, day 1), repeated every 21 days. Then, intensity-modulated radiation therapy (IMRT) was administered: a total dose of 70 Gy to the gross tumor volume (GTV), 66 Gy to involved cervical lymph nodes, 64 Gy to the prophylactic radiation area of the primary lesion, and 58 Gy to bilateral cervical fields (all levels). An extra target volume (a total dose of 70 Gy) was given to the occipital lymph node, and an extra prophylactic dose of 58 Gy was given to the 5- to 10-mm surrounding area. All doses were given in 32 fractions, 5 days per week. The whole course of treatment was completed with no disruptions.

**Fig. 3:**
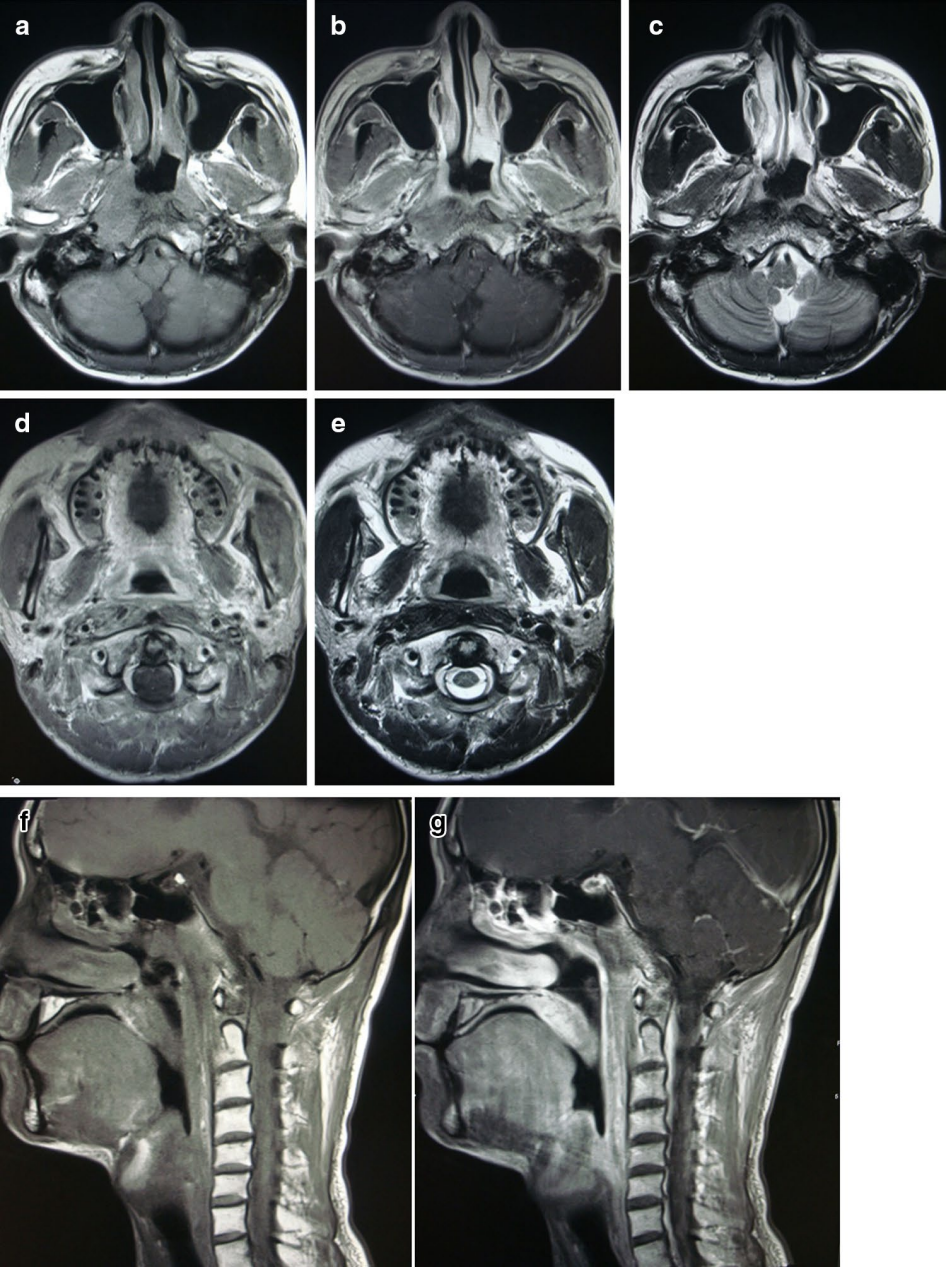
MR imaging of the NPC patient at 3 months after treatment. T1-weighted axial MR image of the nasopharynx **a** without contrast, **b** with contrast, and **c** T2-weighted axial MR image of the nasopharynx show edema signal of the nasopharyngeal mucosa but no mass. T1-weighted axial MR image **d** with contrast of previous occipital lymph node level and **e** T2-weighted axial MR image of previous occipital lymph node level show normal structure now. T1-weighted sagittal MR images **f** without contrast and **g** with contrast reveal no mass as well

Follow-up after completion of radiotherapy showed that the primary lesion and lesions in the cervical lymph nodes and the occipital lymph node could still be detected by MRI; however, they were much smaller than the sizes before treatment. At 1-month follow-up, the primary lesion and lesions in cervical lymph nodes and the occipital lymph node had shrunk further. At 3-month follow-up, no primary lesion or lesions in lymph nodes were detected (Fig. 3). However, 6 months after treatment, the patient returned with a mass of 40 mm × 45 mm in the middle of the sternum. Later, ultrasound-guided biopsy histologically confirmed undifferentiated carcinoma. Further examination of whole-body positron emission tomography/computed tomography revealed multiple fluorodeoxyglucose uptake, with foci in the sternum, left ilium, bilateral internal mammary lymph nodes, and spleen, all of which were considered metastases for the high standard uptake value. The nasopharynx and related regions remained well controlled without sign of recurrence. Other abnormal indexes were EBV VCA-IgA titer of 1:320, EBV EA-IgA titer of 1:20, and EBV-DNA load of 5.51 × 10^3^ copies/mL. Thus, the latest diagnosis of this patient was multiple distant metastases after chemoradiotherapy for NPC. Palliative chemotherapy was needed immediately in this situation, but the patient and his parents refused further treatment and left. Long-term follow-up is being continued.

## Discussion

NPC is an aggressive disease that metastasizes to lymph nodes, mostly the cervical lymph nodes. In an analysis of 924 NPC patients, Mao et al. [6] sought to determine the pattern of cervical lymphatic metastases. In these patients, sentinel metastases were found in the retropharyngeal space and level II, followed by level III, level V, level IV, and supraclavicular area. Another similar study of 779 patients by Chen et al. [7] showed the rates of cervical lymphatic metastases in different levels: the highest was 76.6% in the retropharyngeal space, followed by 64.1% in level IIb, 49.3% in level IIa, 23.6% in level III, 8.6% in level Va, 4.2% in level IV, 2.7% in level Vb, and 0.13% in level I. However, studies of NPC lymphatic metastasis did not reveal a lymphatic drainage pathway to the occipital region. One possible approach for occipital lymph node metastasis from NPC, based on topographic anatomy [8], was presumed as the nasopharynx → jugulodigastric lymph nodes → superior deep lateral cervical lymph nodes → deep lateral cervical lymph nodes → occipital lymph node (backflow). In the present case report, the patient had T4N3M0 NPC with lymphatic metastasis in almost all levels; therefore, the occipital region could presumably be involved from lymph backflow of communicating lymphatic drainage branches.

A remarkable feature of this case was the notably large bilateral cervical lymph nodes, which may have contributed to the occipital lymph node metastasis described above. The tumor of this patient was staged as N3 category; the definition of the tumor in N3 category is “metastasis in a lymph node(s) >6 cm and/or to supraclavicular fossa, the last stage of N category, suggesting the worst prognosis among patients with lymphatic metastasis and a very high risk of distant metastasis.” [5] In this case, we suggested that not only the lymph node size or supraclavicular fossa metastasis but also uncommon regions such as the occipital lymph node was associated with a high risk of distant metastasis. On the other hand, MRI scans of lymph nodes in the neck and occipitalia showed similarities, including approximate T1- and T2-weighted signals, the same liquefactive necrosis signs, and semblable lymphatic fusion. These imaging characteristics suggested that the occipital lymph node is very large and identical to the cervical metastatic lymph nodes. Pathologic confirmation indicated that the origin of the occipital lymph node metastasis was EBER-positive. It is well known that EBV plays an important role in tumorigenesis and development of NPC and has been shown to be a biomarker of NPC [9–12]. Considering these results, we postulate that extensive cervical lymphatic metastasis, especially those meeting the criteria of N3 category, could sporadically cause lymphadenectasis in unusual sites, which we hypothesized as the medium transition between local metastasis and distant metastasis. Lymphadenectasis in unusual sites likely tremendously increases the risk of systemic metastasis. Therefore, for patients who have N3 category tumors, physicians should be aware of unconventional lymphatic metastases, such as metastasis to the occipital lymph node. Once an unusual indication is detected, more extensive analyses should be performed until a final diagnosis is reached.

Metastasis to the occipital lymph node is uncommon. It occasionally occurs in cases of skin cancer or malignancies of the cutaneous appendages of the head and neck, scalp lipoma or liposarcoma, scalp inflammation, lymphoma, malignancies of the external auditory canal, and melanoma of the head and neck. Other rare cases of occipital lymph node metastasis have been seen in sweat gland tumor [13, 14], lung cancer [15, 16], and thyroid cancer [17, 18]. Various treatment strategies for occipital lymph node metastases from cancers other than NPC are shown in Table 2. In a thyroid papillary carcinoma case, reported by Lin et al. [17] in 1997, the primary thyroid papillary carcinoma and the occipital metastatic mass were resected. Without further chemotherapy or radiotherapy, the patient died 17 months later due to seizures caused by metastasis to the brain [17]. The case showed that, although surgery could remove the occipital metastasis, distant metastasis would be a fatal failure. A case reported by Sheth et al. [14] from Memorial Sloan Kettering Cancer Center demonstrated another potentially effective multidisciplinary therapy for occipital metastasis. In this case of mucinous eccrine carcinoma, excision was performed and radiation was administered sequentially to the occipital area and lymph nodes, resulting in 4 years of disease-free survival. Later, two craniotomies and two courses of radiation to the brain and centrums helped the patient live an additional 4 years, after which the disease recurred [14]. This long-term survival was encouraging, reminding us that a promising outcome can be achieved in rare cases of occipital metastasis from different malignancies. The comprehensive method used in common metastases, and multidisciplinary management including surgery and radiotherapy might be effective.

**Table 2:** Occipital lymph node metastases from different diseases

Authors/year	Sex/age	Primary disease	Histology	Treatment	Result
Tian et al. [13]/1992	5 patients:	Absent	Squamous cell carcinoma	Resection	Three survived (median follow-up, 68 months)
	M/30Y,				Survived
	F/40Y,				Died
	M/45Y,				Died
	F/37Y,				Survived
	M/52Y				Survived
Tian et al. [13]/1992	M/22Y	Sweat gland tumor	Syringocarcinoma	Resection	Died
Tian et al. [13]/1992	M/26Y	Absent	Melanoma	Resection	Survived (follow-up, 115 months)
He et al. [15]/1996	F/53Y	Lung cancer	Squamous cell carcinoma	Treatment refusal	Absent
Lin et al. [17] /1997	F/75Y	Thyroid cancer	Thyroid papillary microcarcinoma	Resection	Died after 17 months of treatment
Sheth et al. [14]/2010	F/45Y	Sweat gland tumor	Mucinous eccrine carcinoma	Chemotherapy + radiotherapy	Died 8 years after the first treatment
Kamper et al. [16]/2011	F/69Y	Lung cancer	Bronchial carcinoma	Chemotherapy + radiotherapy	Absent
Karabeir et al. [18]/2011	F/82Y	Thyroid cancer	Thyroid follicular carcinoma	Absent	Absent

*F* female; *M* male; *Y* years

It is well known that radiotherapy is the principal treatment of NPC. Currently, IMRT is widely used because it can maximize the radiation dose to the target and minimize exposure to surrounding critical structures [19], simultaneously increasing the locoregional control rate and decreasing serious adverse effects [20]. In our case, IMRT was administered to the primary lesion, the cervical lymph nodes, and the pathologically confirmed occipital lymph node. The same 70-Gy dose was administered to both the nasopharyngeal neoplasm and the occipital lymph node and resulted in a good local regional control so far, indicating that a standard IMRT dose could achieve satisfying local control; however, in this special case of unconventional lymphatic metastasis of NPC, radiation therapy alone was not enough to prevent distant metastasis. Recently, it was reported that simultaneous integrated boost-intensity modulated radiotherapy (SIB-IMRT) for patients with pediatric and adolescent NPC was an optional treatment. In the study by Tao et al. [21], 34 patients (age 8–20 years) received SIB-IMRT combined with chemotherapy; the results showed that this combination treatment could achieve excellent long-term locoregional control with mild incidence of late toxicities. In these cases, distant metastasis was the primary cause of failure [21], which was consistent with the result of the present case study. On the other hand, as a systemic cure, chemotherapy combined with radiotherapy plays an important role in the treatment of locally advanced NPC. In the case presented here, neoadjuvant chemotherapy followed by concurrent chemoradiotherapy was disappointing in terms of systemic control, since multiple distant metastases occurred in a short time (6 months). Perhaps more aggressive chemotherapy, such as adjuvant chemotherapy, should be given in cases of uncommon lymphadenectasis like occipital lymph node metastasis. Besides radiotherapy and chemotherapy, targeted therapy could be another option. In 2005, Chan et al. [22] launched a multicenter phase II study to investigate whether cetuximab in combination with chemotherapy could benefit NPC patients with recurrence or metastasis. The results were promising: the disease control rate of cetuximab in combination with chemotherapy was 60%, and adverse effects were acceptable.

## Conclusions

Although occipital lymph node metastasis is rare in NPC, it may present in patients with extensive cervical lymph node involvement. Aggressive treatment with combined IMRT and chemotherapy might be beneficial in these cases. In radiotherapy such as IMRT, the occipital lymph node could be treated as another GTV with radical radiation dose. Distant metastasis remains the primary cause of treatment failure. The proper treatment intensity of chemotherapy is unclear in this context; however, multidisciplinary medical management seems necessary. This special case is still open for discussion and comprehensive study.

## Abbreviations

AJCC: American Joint Committee on Cancer
EA: early antigen
EBER: Epstein-Barr virus encoded RNA
EBV: Epstein-Barr virus
GTV: gross tumor volume
FDG: fluorodeoxyglucose
H and E: hematoxylin and eosin
IMRT: intensity-modulated radiation therapy
MRI: magnetic resonance imaging
PET/CT: positron emission tomography/computed tomography
NPC: nasopharyngeal carcinoma
SUV: standard uptake value
VCA: viral capsid antigen
